# Fyn-T Kinase Regulates DHA-Induced Pyroptosis in Immortalized Normal Human Astrocytes

**DOI:** 10.3390/cells14191530

**Published:** 2025-09-30

**Authors:** Ai Ling Cheng, Yuek Ling Chai, Jasinda H. Lee, Clara Y. B. Low, Helen L. Ong, Gavin S. Dawe, Thiruma V. Arumugam, Deron R. Herr, Michelle G. K. Tan, Mitchell K. P. Lai

**Affiliations:** 1 Department of Pharmacology, Yong Loo Lin School of Medicine, National University of Singapore, Singapore 117600, Singaporephcdgs@nus.edu.sg (G.S.D.);; 2 NUS Healthy Longevity Translational Research Programme, Yong Loo Lin School of Medicine, National University of Singapore, Singapore 117456, Singapore; 3 Department of Clinical Translational Research, Singapore General Hospital, Singapore 169856, Singapore; 4 Neurobiology Programme, Life Sciences Institute, Centre for Life Sciences, National University of Singapore, Singapore 117456, Singapore; 5 Department of Microbiology, Anatomy, Physiology and Pharmacology, School of Agriculture, Biomedicine and Environment, La Trobe University, Bundoora, VIC 3086, Australia; 6 Department of Biochemistry, Molecular Biology and Pharmacology, Indiana University School of Medicine—Terre Haute, Terre Haute, IN 47809, USA

**Keywords:** Docosahexaenoic acid, Fyn kinase, immortalized normal human astrocytes, neurodegeneration, neuroinflammation, pyroptosis

## Abstract

Dysregulation of astroglia-mediated neuroinflammation is known to be involved in neurodegenerative diseases. Amongst multiple inflammatory pathways, pyroptosis is characterized by inflammatory cell death following inflammasome activation. Recently, the omega-3 poly-unsaturated fatty acid, DHA, has been identified as a pyroptosis inducer, although the underlying mechanisms remain unclear. In this study, we investigated the role of the alternatively spliced T-isoform of Fyn kinase (FynT) in DHA-induced astroglial pyroptosis. Immortalized normal human astrocytes (iNHA) expressing wild-type FynT (FynT-WT), kinase-dead mutant FynT (FynT-KD), or empty vector (EV) controls were treated with DHA and assessed for pyroptotic activation. We found that DHA-treated FynT-WT cells exhibited significantly reduced cytosolic lactate dehydrogenase release, pyroptotic morphology and markers (cleaved caspase-1 and its substrates, cleaved caspase-3 and gasdermin-D N fragments) compared to either EV or FynT-KD cells. No significant differences in pyroptotic activation were observed between EV and FynT-KD cells. In addition, no differences in immunoreactivities of pro- or anti-apoptotic markers (Bax or Bcl-2) were observed across the DHA-treated cells. In summary, our study postulates a negative regulatory role of FynT kinase in DHA-induced pyroptosis in astrocytes, with implications for further understanding neuroinflammatory mechanisms in neurodegenerative diseases and identification of potential therapeutic targets.

## 1. Introduction

Neuroinflammation refers to inflammatory responses mediated by the brain’s innate immune system, involving the activation of microglia and astrocytes in complex processes accompanied by multiple molecular and cellular changes. Dysregulation of neuroinflammation is now recognized as one of the hallmarks of neurodegenerative diseases such as Alzheimer’s disease (AD) and Parkinson’s disease [1,2,3,4]. In particular, pro-inflammatory factors released by activated astrocytes have been associated with the dysregulation of excitatory neurotransmitters like glutamate, synaptic dysfunction, loss of blood–brain barrier integrity and production of reactive oxygen species (ROS) [2,5]. Of the multiple processes known to contribute to inflammatory responses, pyroptosis is characterized by a lytic inflammatory cell death, which was originally reported to promote rapid clearance of pathogenic microorganisms via the formation of inflammasomes [6]. Subsequent studies found that inflammasome activation could also be primed by endogenous factors including ROS, lipid metabolites, complement and misfolded proteins, leading to observations of pyroptotic processes in a wide range of vascular, metabolic and neuroinflammatory diseases [7,8]. The pyroptotic pathway is characterized by inflammasome-mediated caspase-1 activation, followed by proteolytic cleavage of several proteins into their mature active forms, including pro-inflammatory interleukins IL-1β and IL-18 [9,10], and the N-terminal cleaved fragment of gasdermin D (GSDMD-N) [11]. GSDMD-N oligomerizes to form pores on the cell membrane, allowing influx of water into the cell and causing a characteristic cytoplasmic swelling during pyroptosis [12,13].

Multiple biomolecules are now known to regulate neuroinflammation, including endogenous bioactive lipids, which have also been implicated in the dysregulated neuroinflammation of autoimmune and neurodegenerative diseases [14,15]. Docosahexaenoic acid (DHA), an omega-3 poly-unsaturated fatty acid, has recently been identified as a putative agent that could trigger pyroptosis in microglial cells [16]. Under neurodegenerative conditions, increased levels of DHA are released from membrane phospholipids via phospholipase A2 (PLA2) [17], thus potentially driving a feedback loop which perpetuates the release of DHA whose metabolites may have pro-inflammatory properties, resulting in subsequent activation of pyroptotic neuronal death. While the signalling mechanisms underlying DHA-mediated pyroptosis are largely unclear, recent work has suggested an involvement of Src family non-receptor tyrosine kinases [18,19,20]. One of its members, Fyn kinase, has been implicated in the pathogenesis of AD [21,22] and has also been extensively studied for its role in neuroinflammation [23,24,25]. Fyn is alternatively spliced into two main isoforms, FynB and FynT. Interestingly, while high levels of FynB are endogenously expressed in the brain, only the FynT isoform has been specifically found to be upregulated in AD, PDD and DLB neocortex [3,26]. Furthermore, this increased FynT expression correlated with cognitive impairment, neuropathological burden, as well as markers of microglia and astrocyte activation [3,26]. Additional in vitro studies found that the FynT isoform mediated TNF-induced inflammatory responses in astrocytes [27].

Several studies have reported activation of pyroptosis in microglial cells, including in a DHA-induced cell model [16,28,29,30]. In contrast, pyroptosis activation has not been extensively characterized in astrocytes [31,32]. Given the potential involvement of Fyn kinases in pyroptosis, as well as a FynT-isoform specific regulation of astrocytic inflammatory responses, we aim to investigate the potential role of FynT in modulating DHA-induced pyroptosis in astrocytes. In this study, we used immortalized normal human astrocytes (iNHA) stably transfected with wild-type FynT (FynT-WT) or a kinase-dead mutant (FynT-KD) to investigate the role of FynT kinase in modulating caspase-1/GSDMD-mediated pyroptotic pathway.

## 2. Materials and Methods

### 2.1. iNHA Cells

Immortalized normal human astrocytes (iNHA) are primary human astrocytes stably transfected with constructs encoding E6, E7 and human telomerase reverse transcriptase (hTERT) [33]. The development and characterization of iNHA clones stably expressing pCMV6 vector control (EV), FynT-WT or FynT-KD (with a mutation of lysine to methionine at amino acid position 296) have been previously described [27]. The cells were maintained in High Glucose Dulbecco’s Modified Eagle Medium (DMEM) supplemented with 10% FBS and 1% MEM non-essential amino acids and penicillin-streptomycin (Thermo Fisher Scientific, Waltham, MA, USA) at 37 °C in 5% CO_2_ incubator. Cell cultures were passaged at 80–100% confluence, on average every 3–4 days, and were discarded after a maximum of 20 passages. Mean assay values derived from iNHA were based on three independent experiments, unless otherwise stated.

### 2.2. DHA Preparation

In total, 25 mg of *cis*-4,7,10,13,16,19-Docosahexaenoic acid (DHA) (Sigma-Aldrich, Burlington, MA, USA) was resuspended in 2.5 mL of methanol, then aliquoted into 100 μL tubes. Methanol was evaporated and removed by a speed vacuum concentrator. DHA pellets were purged with nitrogen gas before storage at −20 °C. Prior to treatment, pellets were solubilized in 10% fatty-acid-free bovine serum albumin (Sigma-Aldrich, Burlington, MA, USA) and diluted to the working concentration using DMEM (Thermo Fisher Scientific, Waltham, MA, USA).

### 2.3. 3-(4,5-Dimethylthiazol-2-Yl)-2,5-Diphenyltetrazolium Bromide (MTT) Assays

iNHA cells were plated at a density of 2 × 10^3^ cells/well on 96-well clear-bottom plates for the MTT (Sigma-Aldrich, Burlington, MA, USA) assay to determine cellular metabolic activity and viability while being treated with increasing concentrations of DHA (0, 25, 50, 100, 200, 500 μM) in 100 μL serum-free medium for 4 and 24 h at 37 °C on a shaking water bath and protected from sunlight using aluminum foil-covered lids. At the end of treatment, MTT was added to each well and incubated for 2 h at 37 °C for the detection of the reduced purple formazan product, which was dissolved in 100% dimethyl sulfoxide (Sigma-Aldrich, Burlington, MA, USA). The Biotek Epoch™ microplate spectrophotometer was used to measure absorbance at 550 nm (BioTek, Winooski, VT, USA). To determine the % viability of the treated cells, readouts of untreated (0 μM DHA) iNHA cells were set to 100%.

### 2.4. Morphological Determination of Pyroptotic Cells

Morphological changes in cells treated with DHA were captured for 6 to 8 independent fields per well at hourly time-points using the Nikon Eclipse TS100 (Nikon Instruments Inc., Tokyo, Japan) inverted microscope with a 20× objective. Cells with characteristic cytoplasmic swelling were denoted as ‘pyroptotic’. Intact cells were denoted as ‘normal’. Lysed or dead cells were not included in the cell count. The average number of ‘pyroptotic’ and ‘normal’ cells was determined for each clone and expressed as a percentage of total cells.

### 2.5. Caspase-1 Activity Assays

Caspase-Glo^®^1 kits (Promega, Madison, WI, USA) were used for the quantification of Caspase-1 activity as per the manufacturer’s instructions. iNHA cells were plated onto a black-walled, clear-bottom 96-well plate and subject to DHA treatment. At the end of treatment, Caspase-Glo^®^1 reagent was added in an equivalent volume to the cell culture media and incubated at room temperature for 30 min with gentle shaking. Luminescence readings were recorded on the Biotek Flx800™ microplate fluorescence reader (BioTek, Winooski, VT, USA).

### 2.6. RNA Extraction and qPCR

Cells were harvested and lysed using TRIzol™ reagent (Thermo Fisher Scientific, Waltham, MA, USA). The aqueous phase from the TRIzol™-chloroform mixture post-centrifugation was extracted, followed by the addition of 70% ethanol in a 1:1 ratio. To further minimize potential contamination with genomic DNA, the resulting RNA extract was subject to purification using the Nucleospin^®^ RNA purification kit (Machery-Nagel, Düren, Germany) according to the manufacturer’s instructions. For quantitative real-time polymerase chain reaction (qPCR), RNA samples were converted into cDNA using the High-Capacity cDNA Reverse Transcriptase Kit (Thermo Fisher Scientific, Waltham, MA, USA). Samples were run in duplicate, and the expressions of genes of interest and the GAPDH housekeeping gene were determined using Step One Plus Real Time PCR System with the program settings: 60 °C, 2 min; 95 °C, 10 min; followed by 40 cycles of 95 °C, 15 s and 60 °C, 1 min (Thermo Fisher Scientific, Waltham, MA, USA). Relative gene expression was calculated using the 2^−ΔΔCT^ method. Normalization of the expression of each target gene was carried out by dividing the relative signal intensity of each gene of interest by that of GAPDH. Primers used are listed in Table 1. Four independent experiments were conducted.

### 2.7. Lactate Dehydrogenase (LDH) Release

iNHA cells were plated on a 96-well clear-bottom plate for the determination of LDH release with the CytoTox 96^®^ assay (Promega, Madison, WI, USA) as per the manufacturer’s instructions. Levels of LDH released into medium were expressed as a percentage of the maximum LDH release from lysed cells (positive control using lysis solution, set at 100%). Colorimetric readings were measured using the Biotek Epoch™ microplate spectrophotometer (BioTek, Winooski, VT, USA).

### 2.8. Immunoblotting

iNHA cell lysates were treated with 5% β-mercaptoethanol-containing Laemmli buffer, then heated at 95 °C for 5 min before loading onto 10% SDS-polyacrylamide gels. Proteins were electrophoresed using the Mini-PROTEAN^®^ system (Bio-Rad, Hercules, CA, USA) and transferred onto PVDF membranes using the Invitrogen iBlot^®^ dry blotting system (Thermo Fisher Scientific, Waltham, MA, USA) or the TransBlot Turbo transfer system (only for cleaved caspase-3, GSDMD-N) (Bio-Rad, Hercules, CA, USA). Membranes were blocked in 5% skimmed milk in PBS containing 0.1% Tween-20 (PBS-T) for 1 h before overnight incubation with primary antibodies at specific concentrations (see Supplementary Table S1) in PBS-T at 4 °C. After incubation, membranes were washed three times in PBS-T at 25 °C for 10 min. Thereafter, membranes were incubated with respective horseradish peroxidase (HRP)-conjugated secondary antibodies (1:5000 dilution, Jackson ImmunoResearch, West Groove, PA, USA) for 1 h at 25 °C, then washed three times with PBS-T at 25 °C for 10 min. The blots were visualized using the Luminata™ HRP substrate (Merck Millipore, Billerica, MA, USA) and quantified with the Alliance 4.7 image analyser (UVItec, Cambridge, UK). Immunoblots were re-probed with monoclonal anti-β-actin (Sigma Aldrich, St Louis, MO, USA) at a 1:5000 dilution as a loading control. Three to four independent experiments were conducted.

### 2.9. Statistical Analyses

Statistical analyses and graphing were performed on SPSS (version 26, IBM, Chicago, IL, USA) and Prism (version 7, GraphPad, Boston, MA, USA) software, respectively. Significant differences between the two groups were analyzed using Student’s *t*-tests, while pairwise differences among the groups were analyzed by one-way analysis of variance (ANOVA), followed by post hoc Bonferroni or Dunn’s tests. Analyses of pairwise differences among groups with more than one independent variable were carried out using two-way ANOVA with post hoc Bonferroni or Dunn’s tests. For all analyses, *p*-values of <0.05 were considered to be statistically significant.

## 3. Results

### 3.1. DHA Treatment-Induced Morphological and Molecular Changes Characteristic of Pyroptotic Activation in iNHA

**Figure 1:**
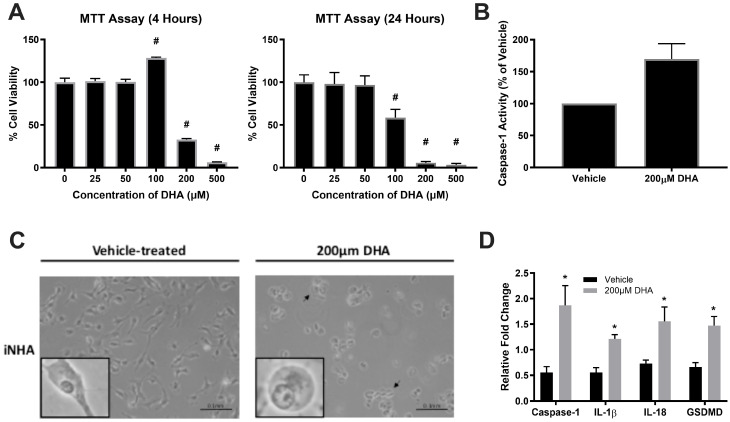
DHA treatment of iNHA cells induced morphological and molecular changes consistent with pyroptotic activation. (**A**) iNHA were incubated with increasing concentrations of DHA for 4 h (left graph) and 24 h (right graph), respectively, and evaluated for cell viability by MTT assay. (**B**) Increased caspase-1 activity was apparent at 4 h after DHA treatment. (**C**) iNHA cells were treated with vehicle or 200 μM DHA for 4 h and monitored for morphological changes by phase-contrast microscopy, with higher-magnification insets of individual cells to illustrate changes characteristic of pyroptotic cytoplasmic swelling (also indicated by black arrows in the representative micrograph on the right, scale bar: 0.1 nm). (**D**) Gene expression of key pyroptotic mediators, including caspase-1, IL-1β, IL-18 and gasdermin D (GSDMD), was determined by qPCR at 2 h after vehicle (black bars) or DHA treatment (grey bars). ^#^ *p* < 0.05 compared to 0 time-point (one-way ANOVA followed by post hoc Dunn’s tests). * *p* < 0.05 compared to vehicle control (Student’s *t*-tests).

iNHA cell viability is affected by DHA treatment in a dose-dependent manner, with significantly reduced viability at high (200 and 500 μM) but not low doses (25 and 50 μM) of DHA after 4 h or 24 h of DHA treatment (Figure 1A), consistent with previous findings in BV2, a microglial cell line [16]. Upon 200 μM DHA treatment, iNHA showed increases in caspase-1 activity (Figure 1B), and a subset of cells exhibited distinct cytoplasmic swelling 4 h post-treatment (Figure 1C), similar to the pyroptotic morphological changes found in BV2 cells (Supplementary Figure S1), thus further supporting pyroptosis activation by high-dose DHA. The effects of DHA were unlikely to be mediated by any changes in endogenous expression of either FynT or FynB over the time-course of our experiments (Supplementary Figure S2). Furthermore, gene expressions of key pyroptotic markers (caspase-1, interleukins IL-1β, IL-18 and GSDMD) were measured by qPCR at 2 h post-treatment, and were all found to be significantly upregulated (Figure 1D), consistent with previous findings of gene expression changes in DHA-induced pyroptosis [16].

### 3.2. Kinase Activity of FynT Suppressed the Activation of DHA-Induced Pyroptosis and Generation of Cleaved Caspase-3 in iNHA

**Figure 2:**
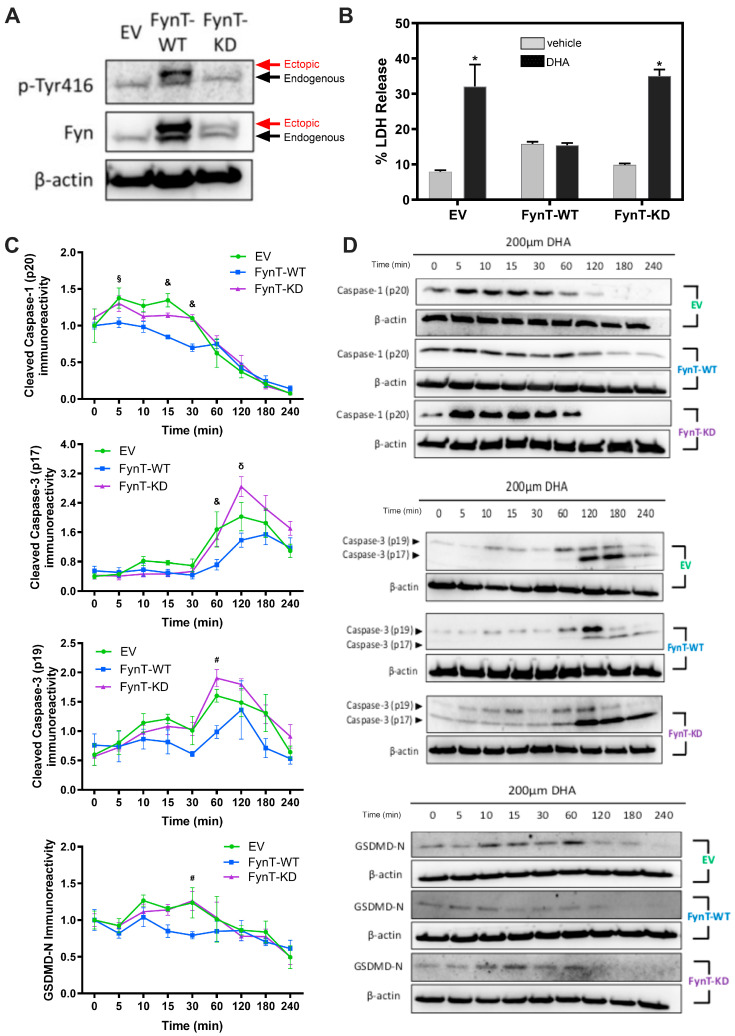
FynT activity negatively regulates pyroptotic markers in iNHA. (**A**) Representative immunoblots of iNHA clones stably expressing EV, FynT-WT or FynT-KD were assessed for Fyn immunoreactivity and Fyn kinase activity using antibodies against Fyn and pTyr416, respectively. Ectopic (transfected) expression of Fyn is denoted by the red arrow (upper band), and endogenous Fyn is denoted by the black arrow (lower band). The kinase-dead effect of transfected FynT in FynT-KD was confirmed by the presence of ectopic bands corresponding to Fyn in both FynT-WT and FynT-KD, but by the loss of an ectopic band corresponding to pTyr416 in FynT-KD. All the iNHA clones were treated with 200 μM DHA and compared for clone-specific changes in the immunoreactivities of pyroptotic markers for up to 4 h. (**B**) LDH release (% of the maximum LDH release from lysed cells) was measured at 4 h post-treatment. (**C**) Graphs depicting time-course and (**D**) representative immunoblots of cleaved caspase-3 (p19 and p17), cleaved caspase-1 (p20) and GSDMD-N across a 4 h time-course. Data presented were derived from 3 to 4 independent experiments. Values are expressed as mean ± S.E.M. * *p* < 0.05 when compared to vehicle control or 0 time-point using Student’s *t*-tests. For time-course experiments, significant differences were observed between clones using two-way ANOVA followed by post hoc Bonferroni’s tests, where: ^&^ *p* < 0.05 when comparing EV and FynT-KD to FynT-WT; ^δ^ *p* < 0.05 when comparing EV and FynT-WT to FynT-KD; ^§^ *p* < 0.05 when comparing EV to FynT-WT; ^#^ *p* < 0.05 when comparing FynT-KD to FynT-WT.

Upon validating pyroptotic activation in the DHA-treated iNHA cells, we sought to determine whether FynT kinase is implicated in this DHA-induced pyroptosis pathway. This was achieved by comparing the activation of pyroptotic markers between the three iNHA clones (EV, FynT-WT and FynT-KD). Immunoblot analyses confirmed the ectopic (transfected) expression of FynT in each clone (upper band, as indicated by red arrows in Figure 2A), with an increase in Fyn kinase activity in FynT-WT clone, and little or no Fyn kinase activity in FynT-KD, as determined by pTyr416 (an autophosphorylation site of Src-family kinases, including Fyn, known to activate enzymatic activity [34]) immunoreactivities of the upper bands (Figure 2A).

To monitor whether FynT kinase activity may modulate key molecules of pyroptosis, including cleaved caspase-1 (p20) and GSDMD-N, as well as cleaved caspase-3 (p19 and p17), which is known to regulate both pyroptosis and apoptosis, immunoreactivities of corresponding cleaved fragments were compared across a 4 h timeframe post-DHA treatment in all three iNHA clones (EV, Fyn-KD, Fyn-WT). Release of LDH indicates cell-damaging processes or cytotoxicity resulting in the loss of plasma membrane integrity, including necrosis, apoptosis and pyroptosis. Figure 2B shows, consistent with an induction of pyroptosis, increased LDH release in vector-control EV cells with DHA treatment, an effect which was abrogated in the FynT-WT clones. Interestingly, LDH release was restored in FynT-KD cells to levels similar to those of the EV controls. For pyroptotic markers, Figure 2C,D show that with DHA, levels of cleaved caspase-1 were significantly increased in EV and FynT-KD at the 15 min and 30 min time-points before rapidly decreasing, while significant changes were not observed in FynT-WT post-DHA treatment. Similar increases in the immunoreactivities of cleaved caspase-3 (both p19 and p17 subunits) and GSDMD-N were evident at later time-points for EV and FynT-KD, again with no corresponding change in FynT-WT. Taken together, our findings suggest that the kinase activity of FynT exerts an inhibitory effect on DHA-induced increases in immunoreactivities of pyroptotic markers at various time-points and may also be protective against pyroptosis-related plasma membrane damage.

### 3.3. Pharmacological Inhibition of Fyn Kinase Increases DHA-Induced Cytotoxicity

**Figure 3:**
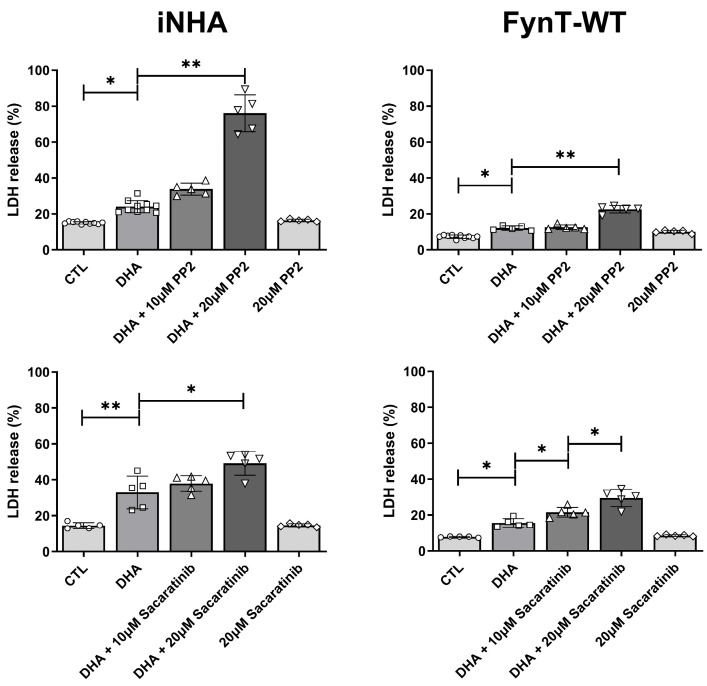
Fyn kinase inhibitors exacerbate DHA-induced LDH release in iNHA. Untransfected iNHA (**left column**) or FynT-WT clones (**right column**) were treated with 350 μM DHA with or without overnight pretreatment with PP2 or sacaratinib dissolved in DMSO at the concentrations indicated. Bar graphs show mean ± SD LDH release (% of the maximum LDH release from lysed cells), measured at 4 h post-DHA treatment. CTL group was treated with vehicle only (0.1% DMSO), while DHA group was treated with DHA and vehicle. * *p* < 0.05 or ** *p* < 0.01 significant pairwise comparisons using ANOVA with Bonferroni post hoc tests, based on a minimum of *n* = 5 independent measurements.

To corroborate a protective effect of Fyn kinase as described above, iNHA cells were subject to co-treatment of DHA with PP2 (Sigma-Aldrich, Burlington, MA, USA) or saracatinib (MedChemExpress, Monmouth Junction, NJ, USA), both potent and selective inhibitors of Fyn kinase [35,36]. Figure 3 shows that pharmacological inhibition of Fyn kinase by PP2 or sacaratinib further exacerbated DHA-induced LDH release in a dose-dependent manner, suggesting a protective role of Fyn kinase against DHA-associated cytotoxicity. PP2 and sacaratinib treatment resulted in similar effects on DHA-treated FynT-WT expressing iNHA cells, although LDH release was generally more repressed compared to untransfected cells, further supporting the cytoprotective effects of FynT.

### 3.4. Kinase Activity of FynT Suppressed Pyroptotic Morphological Changes in DHA-Treated iNHA

**Figure 4:**
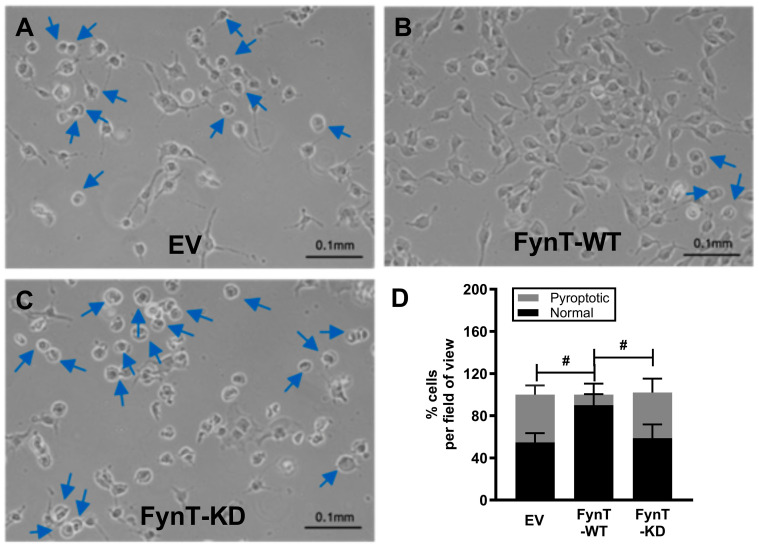
Representative images of iNHA (**A**) EV, (**B**) FynT-WT and (**C**) FynT-KD cells treated with 200 μM DHA for 2 h and monitored for morphological changes by phase-contrast microscopy at ×20 magnification. Blue arrows indicate cells exhibiting cell swelling, a characteristic of pyroptosis. (**D**) The stacked bar graphs display the average percentage of cell counts per field for each iNHA clone, with the black bars depicting normal cells and the grey bars depicting pyroptotic cells. ^#^ Significant difference in % pyroptotic cells between groups (*p* < 0.05, one-way ANOVA with Bonferroni post hoc tests).

Earlier, we demonstrated the gene expression profile and cytoplasmic swelling in iNHA, which are characteristic of pyroptosis upon treatment with DHA (see Figure 1C,D), and we assessed these morphological changes in the iNHA clones (Figure 4A–C) to see whether they are altered in parallel with other pyroptotic markers. We selected the 2 h time-point for these assessments, as caspase-3 subunits are found to be maximally activated between 1 and 2 h, with caspase-1 and GSDMD-N activation occurring prior to this time interval (see Figure 2C), whereas by the 3 to 4 h time-points, pyroptotic markers have returned to basal levels (or lower than basal level in the case of caspase-1), potentially resulting in a floor effect, where differences were not discernible due to clustering at the lower detection limit. Figure 4D shows that, similar to other pyroptotic markers, the proportion of cells exhibiting pyroptotic morphological changes was significantly reduced in FynT-WT compared to both the EV and FynT-KD cells, again suggestive of a protective effect of FynT kinase activity on DHA-induced pyroptosis.

### 3.5. FynT Kinase Does Not Alter Apoptotic Markers Bax and Bcl-2 in DHA-Treated iNHA

**Figure 5:**
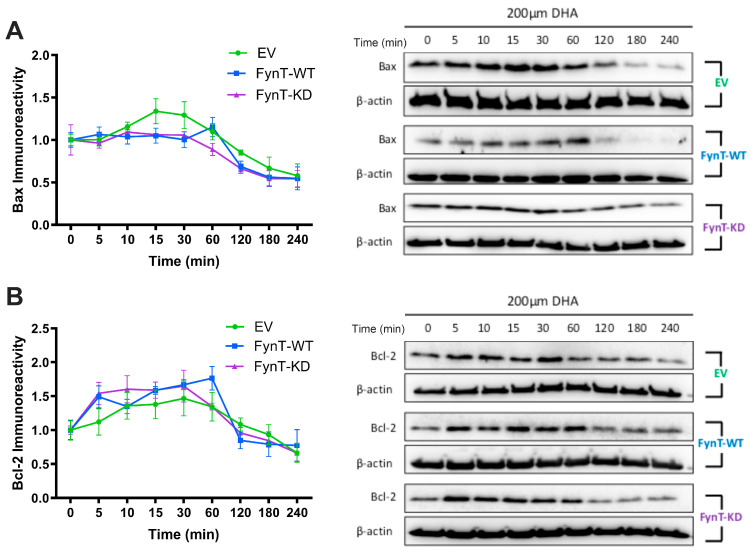
FynT kinase does not alter apoptotic markers in DHA-treated iNHA. Graphs depicting time-course and representative immunoblots of (**A**) Bax and (**B**) Bcl-2 across a 4 h time-course post-DHA treatment. Data presented were derived from 4 independent experiments. Values are expressed as mean ± S.E.M.

Finally, since caspase-3 activation is implicated in both pyroptosis and apoptosis [37,38,39,40], we performed immunoreactivity measurements of pro-apoptotic Bax and anti-apoptotic Bcl-2, and showed that there were no significant differences in the levels of both proteins over 4 h post-DHA treatment in EV, FynT-WT and FynT-KD cells (Figure 5A,B).

## 4. Discussion

Besides their well-established roles in homeostatic and metabolic support for neurons, there has been increasing recognition that astrocytes are crucially involved in the regulation of neuroinflammation, whose dysfunction has been implicated in AD and related neurodegenerative diseases [41,42]. In this context, pyroptosis, an inflammatory programmed cell death initiated by inflammasome formation, may contribute to the neurodegenerative process [43]. Indeed, markers of pyroptosis have been detected in both microglia and astrocytes in AD [44], together with significant increases in cleaved NLRP3-associated cleaved caspase-1 [45] and GSDMD [46]. However, the upstream signals and underlying pathways of pyroptosis in AD and related neurodegenerative diseases remain unclear. We previously showed that high (but physiologically relevant) doses of DHA can activate pyroptosis, likely via the generation of pro-inflammatory DHA metabolites, in a microglia line [16], and have now extended this observation to the human astrocyte-derived iNHA cell line. Furthermore, using iNHA stably transfected with wild-type and kinase-dead mutant FynT, we have demonstrated, for the first time, a potential negative regulatory effect of FynT on molecular and morphological markers of DHA-induced pyroptosis.

**Figure 6:**
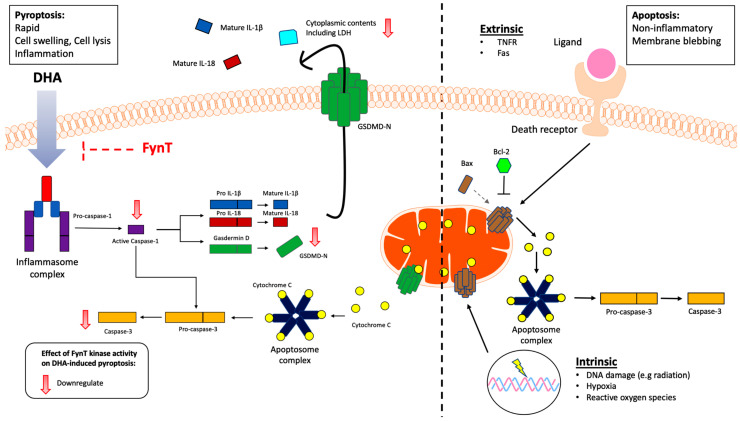
Schematic overview of the putative inhibitory role of FynT kinase activity in modulating DHA-induced pyroptosis and downstream caspase-3 activation in astrocytic cells. Our findings suggest FynT involvement in the DHA-induced caspase-1/GSDMD-mediated pyroptotic process, which may also negatively modulate the downstream activation of apoptotic caspase-3. This pathway is likely distinct from the mitochondrial apoptotic pathway, which involves the mitochondrial apoptotic markers Bax and Bcl-2 in astrocytes. FynT involvement in caspase-3-mediated apoptosis is suggested by our results, but requires confirmatory follow-up studies.

With regard to the time-course experiments, our observations of DHA-induced cleavage of caspase-1 occurred as early as 5 min, followed by GSDMD-N around 30 min and subsequent cleavage of caspase-3 at the 1–2 h time-points, before the markers returned to baseline levels (or below baseline in the case of cleaved caspase-1 and GSDMD-N, see Figure 2C) by 3–4 h, are consistent with the rapid process of classical caspase 1-mediated pyroptosis, which in turn activates downstream caspase 3 [38]. The alternate pathway, where the pore-forming GSDMD-N oligomerizes and mediates the release of cytochrome C from mitochondria, leading to downstream activation of caspase-3 [47], is also congruent with our time-course results. Even though the apoptotic markers Bax and Bcl-2 were unchanged amongst the iNHA clones (Figure 3), our findings that the apoptosis executor caspase-3 was similarly suppressed along with cleaved caspase-1 and GSDMD-N in the FynT-WT cells suggest that FynT may negatively regulate both pyroptosis and apoptosis, the latter independent of Bax/Bcl-2. However, Taabazuing et al. [38] have identified a role of caspase-3 in suppressing the release of GSDMD-N from GSDMD, thus inhibiting activation of pyroptosis. Therefore, the cleavage and activation of caspase-3 that occur with slower kinetics and after the activation of the caspase-1/GSDMD-mediated pyroptosis may serve as a counter-regulatory feedback mechanism on pyroptotic activation to aid in dampening inflammation. Furthermore, caspase-3 is also known to regulate pyroptosis in a pathway involving another member of the gasdermin family, GSDME [48]. Our results, therefore, highlight the complex regulatory relationships amongst components of the pyroptotic pathway, as well as the bidirectional crosstalk between pyroptosis and apoptosis. Figure 6 provides a schematic overview of how FynT kinase may putatively regulate DHA-induced pyroptosis based on this study’s findings.

### 4.1. Implications of Study Results in AD and Related Neurodegenerative Diseases

Using postmortem brain measurements, rodent models and in vitro studies, we have previously found specific upregulation of FynT in both AD and Lewy body dementias, which correlated with reactive astrogliosis, increased levels of pro-inflammatory markers and neuropathological burden [3,26,27]. This led us to propose FynT as an anti-neuroinflammatory therapeutic target for AD [27]. Considering the current results, we speculate that upregulation of FynT may have additional effects, specifically in negatively regulating pyroptosis (via reductions in activated caspase-1, GSDMD and caspase-3), and potentially apoptosis as well (via reduction in activated caspase-3 in a Bax- and Bcl-2-independent manner). The extent to which this negative regulation of programmed cell death occurs in astrocytes undergoing reactive gliosis may then explain their persistence in AD and related neurodegenerative diseases, thereby contributing to the chronic, dysregulated neuroinflammation characteristic of these conditions. Interestingly, there are analogous examples in oncology where the loss of caspase activity in cancer cells, via pharmacological inhibition or mutation, inhibits programmed cell death [49,50], leading to resistance to cytotoxic drugs. Therefore, the current study provides additional impetus to investigate FynT as a therapeutic target in AD and related neurodegenerative diseases by countering the inhibition of programmed cell death and aiding the removal of chronically reactive gliotic cells.

### 4.2. Limitations and Research Gaps

There are several limitations and research gaps in the current study which need to be highlighted and followed-up on in future work. Firstly, our in vitro experiments suggest a negative regulatory role of FynT on caspase-1, GSDMD and caspase-3, but the molecular mechanisms underlying this regulatory effect are at present unclear. Since the kinase activity of FynT appears to be important for the regulatory effect, future work should characterize potential phosphorylation substrates of FynT that may be involved in the pyroptotic/apoptotic pathway. Furthermore, due to the inherent variability of the transfection experiments, FynT-KD clones expressed the kinase-dead form of Fyn at a lower level than FynT-WT. Therefore, while the negative regulation of Fyn on pyroptosis is still supported by our data, we were not able to unambiguously attribute the finding to the kinase activity per se, as it remains a possibility that certain non-enzymatic aspects of Fyn may underlie the observed differences. Similarly, while the Fyn kinase chemical inhibitor experiments further supported protective effects of Fyn against DHA-induced cytotoxicity (see Figure 3), we cannot unreservedly ascribe the effect specifically to Fyn, as both inhibitors also affect other Src family kinases [35,36]. Secondly, while our study was primarily focused on FynT effects on pyroptosis, and we have reported on multiple pyroptotic markers, the finding of negative regulation of the apoptosis executor caspase-3 suggests, as others have, a crosstalk between pyroptosis and apoptosis (see Section 4.1 above), and future work should more comprehensively characterize potential FynT effects on apoptotic pathways, as well as potential effects of caspase-3 on pyroptotic pathways. In terms of the generalizability of the present findings, follow-up studies should include FynT effects in other inflammatory cell types (e.g., microglia) and explore other activators of pyroptosis besides DHA and their metabolites. Additionally, while our findings are based on immortalized normal human astrocytes (iNHA), which are reported to have close phenotypic resemblance to primary astrocytes [27,33], they are nevertheless cell lines, and we cannot exclude potential clonal effects of the FynT clones used. With regard to potential clonal effects, the generation of FynT knockout cells and follow-up experiments will provide further support for its regulatory role on pyroptosis. Furthermore, it is unclear whether the levels of FynT-WT overexpression observed in our cells have any pathophysiological relevance, although we have shown several-fold upregulation of FynT in AD brains [3], similar to changes observed in the cell lines. This, as well as the clonal effect, may be addressed in future work based on animals, for example, with FynT transgenic or knockdown mice. Lastly, while our initial observations on specific FynT changes in AD led us to focus on FynT’s putative involvement in pyroptosis regulation in the current study, whether FynB might also have similar or distinctive regulatory roles is at present unknown and needs to be further investigated.

## 5. Conclusions

In summary, this study reports in vitro experimental data suggestive of a novel FynT-dependent negative regulation for DHA-induced, caspase-1/GSDMD-mediated pyroptotic pathway. A similar suppressive effect on caspase-3 suggests that FynT may regulate apoptotic pathways as well, pending confirmatory follow-up studies. From a pathophysiological perspective, this provides a mechanism underlying the persistence of chronic neuroinflammation found in AD and related neurodegenerative diseases, as the previously observed upregulation of FynT during reactive astrogliosis may suppress programmed cell death in activated pro-inflammatory astrocytes. Our findings, therefore, support FynT inhibition or downregulation as a potential anti-neuroinflammatory therapeutic target for neurodegenerative diseases. However, further studies are needed to validate these in vitro findings.

## Figures and Tables

**Table 1:** qPCR primers used in this study.

Name	Forward Primer Sequence (5′ to 3′)	Reverse Primer Sequence (5′ to 3′)	Amplicon Size
Caspase-1	TTTCCGCAAGGTTCGATTTTCA	GGCATCTGCGCTCTACCATC	54
IL-1β	AGCCAGGACAGTCAGCTCTC	AAGCGGTTGCTCATCAGAAT	171
IL-18	TGCCAACTCTGGCTGCTAAA	TTGTTGCGAGAGGAAGCGAT	104
GSDMD	GTGTACGTGGTGACTGAGGT	CCTCTGCTTCTTATCCGGGA	246
GAPDH	TGACATCAAGAAGGTGGTGAAG	TTACTCCTTGGAGGCCATGTG	241

## Data Availability

The original contributions presented in the study are included in the article/Supplementary Material; further inquiries can be directed to the corresponding authors.

## Supplementary Materials

The following supporting information can be downloaded at: https://www.mdpi.com/article/10.3390/cells14191530/s1, Table S1: List of primary antibodies and dilution factors for immunoblotting in this study; Figure S1: DHA-induced morphologic changes in the BV2 microglial cell line; Figure S2: Effects of DHA treatment on Fyn-T and Fyn-B expression; Supplementary Information: Raw immunoblots for figures.
