# Supplementary material for: Fyn-T Kinase Regulates DHA-Induced Pyroptosis in Immortalized Normal Human Astrocytes
Source: Cells. 2025 Sep 30;14(19):1530. doi: 10.3390/cells14191530 (PMC12523382; doi:10.3390/cells14191530)
Supplement: Supplementary file 1 [file cells-14-01530-s001.zip › cells-3905942-supplementary.pdf]

Supplementary Information

Table S1. List of primary antibodies and dilution factors for immunoblotting in this study

Antibody (Clone name) [Catalogue no.]	Dilution	Species	Company
Anti Fyn antibody (Fyn-01) [ab1881]	1:1000	Mouse mAb	Abcam
Phospho-Tyr416 Src Family [#2101]	1:1000	Rabbit pAb	CST
Cleaved Caspase-1, Asp296 [#67319]	1:1000	Rabbit mAb	CST
Cleaved Gasdermin D, Asp275 (E7H9G) [#36425]	1:500	Rabbit mAb	CST
Cleaved Caspase-3 [#9661]	1:500	Rabbit pAb	CST
Bax [#2772]	1:1000	Rabbit pAb	CST
Bcl-2 (124) [#15071]	1:1000	Mouse mAb	CST
β-actin (AC-74) [A2228]	1:5000	Mouse mAb	Sigma

Abbreviations: Abcam: Abcam plc (Cambridge, UK); CST: Cell Signaling Technology (Danvers, MA USA); mAb: monoclonal antibody; pAb: polyclonal antibody; Sigma: Sigma-Aldrich, Merck Group (Darmstadt, Germany)

Figure S1. DHA-induced morphologic changes in the BV2 microglial cell line

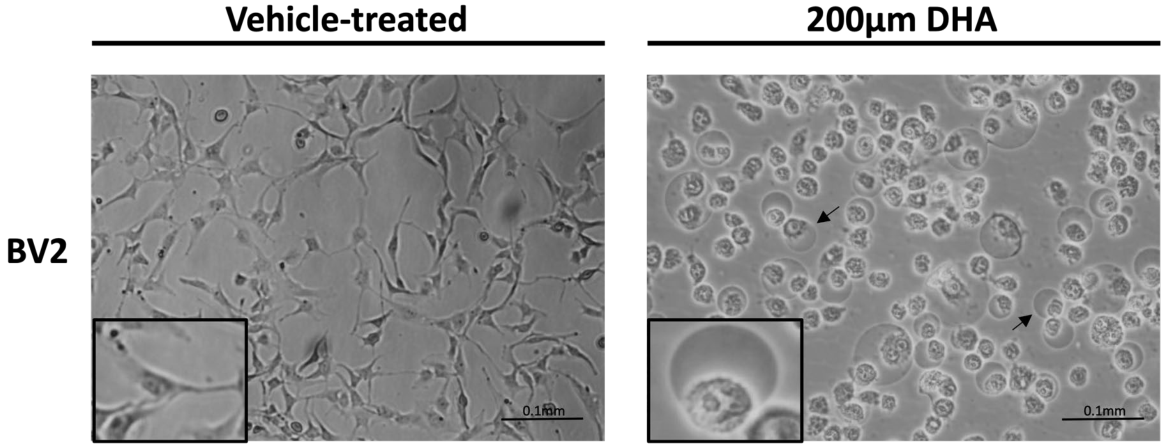

Figure S1. BV2 cells were treated with vehicle or 200μM DHA for 4h and monitored for morphological changes by phase contrast microscopy at x20 magnification. Arrows denoting some of the cells exhibiting cytoplasmic swelling characteristic of pyroptosis. Higher magnification images of a normal, live cell and a pyroptotic cell are shown in lower left inserts of the vehicle-treated and DHA-treated images respectively.

Figure S2. Effects of DHA treatment on Fyn-T and Fyn-B expression

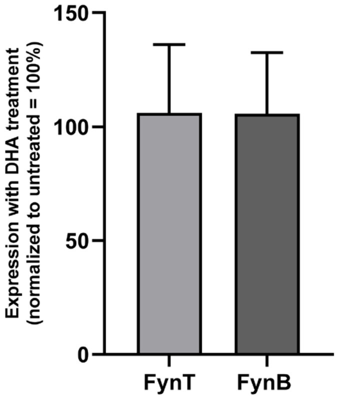

Name	Forward (5’ to 3’)	Reverse (5’ to 3’)	Size (bp)
FynB-specific	TTTCCGCAAGGTTCGATTTTCA	GGCATCTGCGCTCTACCATC	166
FynT-specific	AGCCAGGACAGTCAGCTCTC	AAGCGGTTGCTCATCAGAAT	136

Figure S2. Potential effects of DHA treatment (200 or 300μM) on endogenous expression of FynT and FynB in iNHA cells after 4h were investigated using quantitative polymerase chain reaction (qPCR), with inset showing the forward and reverse primer sequences used (see Methods section of main manuscript for details of qPCR protocol). Relative expression of FynT and FynB was expressed as a percentage (± SD) of untreated iNHA, set at 100%. Bar chart represents n = 3 independent experiments.
